# Pathogen Profile of Children Hospitalised with Severe Acute Respiratory Infections during COVID-19 Pandemic in the Free State Province, South Africa

**DOI:** 10.3390/ijerph191610418

**Published:** 2022-08-21

**Authors:** Ayodeji E. Ogunbayo, Milton T. Mogotsi, Hlengiwe Sondlane, Kelebogile R. Nkwadipo, Saheed Sabiu, Martin M. Nyaga

**Affiliations:** 1 Next Generation Sequencing Unit and Division of Virology, Faculty of Health Sciences, University of the Free State, P.O. Box 339, Bloemfontein 9300, South Africa; 2 Department of Biotechnology and Food Science, Durban University of Technology, P.O. Box 1334, Durban 4000, South Africa

**Keywords:** severe acute respiratory infections, waves of COVID-19 pandemic, respiratory viruses, children, QIAstat-Dx^®^ Respiratory SARS-CoV-2 Panel

## Abstract

Severe acute respiratory infections (SARI) contribute to mortality in children ≤5 years. Their microbiological aetiologies are often unknown and may be exacerbated in light of coronavirus disease 19 (COVID-19). This study reports on respiratory pathogens in children ≤5 years (n = 84) admitted with SARI during and between the second and third waves of COVID-19 infection in South Africa. Nasopharyngeal/oropharyngeal swabs collected were subjected to viral detection using QIAstat-Dx^®^ Respiratory SARS-CoV-2 Panel. The results revealed viral positivity and negativity detection rates of 88% (74/84) and 12% (10/84), respectively. Of the 21 targeted pathogens, human rhinovirus/enterovirus (30%), respiratory syncytial virus (RSV; 26%), and severe acute respiratory syndrome coronavirus 2 (24%) were mostly detected, with other viruses being 20% and a co-infection rate of 64.2% (54/84). Generally, RSV-positive samples had lower Ct values, and fewer viruses were detected during the third wave. Changes in the circulation patterns of respiratory viruses with total absence of influenza virus could be attributed to measures against COVID-19 transmission, which may result in waned immunity, thereby increasing susceptibility to severe infections in the following season. High viral co-infection rate, as detected, may complicate diagnosis. Nonetheless, accurate identification of the pathogens may guide treatment decisions and infection control.

## 1. Introduction

The World Health Organization (WHO) defines patients with severe acute respiratory infections (SARI) as anyone with acute respiratory infection (ARI) with symptoms such as cough and measured fever of ≥38 °C within 10 days of presentation and requiring hospitalisation [1]. In children, SARI is a common cause of morbidity worldwide [2,3] and a leading infectious cause of death in those less than five years of age [3,4]. Globally, in 2019, ARI was estimated to result in at least 740,000 deaths in children under five years of age [5]. Several viral agents, such as influenza virus (IFV), respiratory syncytial virus (RSV), parainfluenza virus (PIV), and human rhinovirus (HRV), account for about 35–87% of cases in children with SARI [6,7]. Viral respiratory co-infections have also been detected in 4–60% of children hospitalised with SARI and may suggest an increased risk for clinical outcome [7,8,9,10].

Viral and bacterial aetiologies of SARI cannot be distinguished based on clinical presentations, and this necessitates specific laboratory diagnoses which may not be available/not routinely performed in some settings. As such, aetiologies of respiratory tract infections (RTIs) are often unknown [11,12,13]. Although, the contribution of viruses to RTIs morbidity and mortality is becoming increasingly evident due to increased availability of better diagnostics technology [14]. Nonetheless, studies suggest that many cases of SARI are still diagnosed empirically and with supportive care in the absence of facilities for diagnosing viruses [15,16]. However, even when presented with the capacity for diagnosis, broad pathogen detection remains challenging, especially in low resource settings [15,17]. In essence, the identity of the viral causative agent/s in many SARI cases remains unknown [18].

Of note, in the absence of resources for testing, clinicians may be inclined to choose early empirical antibiotic treatment in SARI patients before identifying the causative pathogen(s) [19]. Although this practice plays a significant role in controlling disease progression, there are risks that it could also lead to inappropriate use of antibiotics, drug toxicity, and resistance [20,21]. With the advent of rapid multiplex real-time PCR (mRT-PCR) assay for broad pathogen detection, diagnosing specific respiratory viral infection is becoming easier and can help in proper patient management. In addition, prompt viral identification may help initiate an antiviral drug where applicable, enhance supportive therapy [15,16,17], identify possible outbreak, improve infection control, decrease unnecessary use of antibiotics, and ultimately reduce healthcare costs [22]. Imperatively, in an era of unexpected coronavirus disease 2019 (COVID-19) pandemic, there has never been a better time that necessitates the accurate diagnosis of RTIs in children, given the number of cases that could have gone untested [23,24]. While the prompt detection of viral pathogens mostly implicated in SARI in children is important to effectively guide clinical and epidemiological decisions as stated, pharmaceutical interventional measures, such as vaccination, are equally imperative [25]. However, there are currently no licensed vaccine products for RSV and other viral respiratory pathogens. Only influenza vaccination is available yearly as flu shots for children from six months and above in preventing severe influenza cases [26].

Furthermore, the traditional circulating pattern of viral respiratory pathogens since the onset of COVID-19 has drastically changed from previous years due to nonpharmaceutical interventions (NPIs) taken against COVID-19 [27,28,29,30]. These NPIs include social distancing, closure of schools and institutions of higher learning, hand sanitising, restrictions on public gatherings, and wearing of face masks, all of which could have impacted the pathogen spectrum of RTIs in children [27,28,30]. Detection of such changes in the pathogen spectrum can help in outbreak identification and guide the implementation of control strategies [15,31]. Hence, using the QIAstat-Dx Respiratory SARS-CoV-2 Panel (Qiagen Hilden, Germany) (an mRT-PCR test that simultaneously detects and identifies multiple respiratory viral and bacterial nucleic acids in nasopharyngeal swabs (NPS) obtained from individuals suspected of RTIs) [32,33,34], we investigated the profiles of respiratory pathogens in children hospitalised for SARI in the Free State Province, South Africa, during and between the second and third waves of COVID-19 in the country. This was performed with a view to elucidate on changes in the dynamics of circulating viral respiratory pathogens during this period and to identify possible need for future epidemiological surveillance of other viral respiratory pathogens post-COVID-19 era.

## 2. Materials and Methods

### 2.1. Study Design and Setting

This cross-sectional study was conducted at Botshabelo District Hospital, National District Hospital, and Pelonomi Regional Hospital, Free State Province, South Africa, between December 2020 and August 2021. Sampling and participant enrolment took place during the second wave, the period between (time between the second and third waves), and during the third wave of the COVID-19 infection (Table 1).

### 2.2. Definition of the COVID-19 Waves

The waves were defined as the period during which COVID-19 weekly incidence in the Free State was greater than 30 cases per 100,000 persons until the weekly incidence was equal to/or below 30 cases per 100,000 persons (Table 1) [35].

### 2.3. Patient Enrolment

Between December 2020 and September 2021, a total of 149 children with SARI were recruited. Of these, a total of 84 children (n = 28 per waves and period between) whose samples were collected during the weeks of the COVID-19 waves (second and third waves) and period in between the waves were included in this study. These participants were selected to have an even distribution during the periods. The WHO case definitions for SARI were implemented as children admitted with ARI, with a measured fever ≥38 °C and cough, with onset within the last 10 days. Before admission, a structured questionnaire was used to collect patient demographics and specific clinical signs/symptoms.

### 2.4. Specimen Collection

Upon admission, nasopharyngeal and oropharyngeal swabs (BD Diagnostics, Franklin Lakes, NJ, USA) were collected per child by qualified medical personnel. The swabs were inserted in a viral transport media (VTM) (BD Diagnostics, Franklin Lakes, NJ, USA). Samples were labelled and transported to the University of the Free State-Next Generation Sequencing (UFS-NGS) Unit, Division of Virology, Bloemfontein, Free State, South Africa, via cold chain transportation. Samples were registered and given a unique identification number before storage at −80 °C until processing.

### 2.5. Pathogen Analysis

Pathogen detection was performed using QIAstat-Dx Respiratory SARS-CoV-2 Panel (Qiagen, Hilden, Germany). The panel detects, generates cycle threshold (Ct) value, and differentiates nucleic acid from severe acute respiratory syndrome coronavirus 2 (SARS-CoV-2) and the following organism types and subtypes: adenovirus, coronavirus 229E, coronavirus HKU1, coronavirus NL63, coronavirus OC43, SARS-CoV-2, human metapneumovirus A+B (HMPV), influenza A, influenza A H1, influenza A H3, influenza A H1N1/pdm09, influenza B, PIV 1, PIV 2, PIV 3, PIV 4, rhinovirus/enterovirus, RSV A+B, *Bordetella pertussis*, *Chlamydophila pneumoniae*, and *Mycoplasma pneumoniae* [36]. The panel cartridge includes a titered MS2 bacteriophage, which is used as internal control. A positive signal for the internal control indicates that all processing steps performed by the panel cartridge were successful. The assay was performed as per the manufacturer’s instructions. Briefly, 300 µL of the respiratory sample in VTM (BD Diagnostics, USA) was loaded into a QIAstat-Dx RP test cartridge (Qiagen, Hilden, Germany). The test cartridge’s barcode and the corresponding sample’s barcode were scanned by the QIAstat-Dx operational module (Qiagen, Hilden, Germany), and then loaded into the QIAstat-Dx analyser module (Qiagen, Hilden, Germany). The panel was run for 40 cycles and, after approximately 70 min, the results from the targeted pathogens were reported as either positive (with corresponding cycle threshold (Ct) values) or not detected.

### 2.6. Statistical Analysis

Raw data were calculated using Microsoft Excel 365 version 2206, and results are presented as simple percentages, mean, and standard deviations of replicate determinations.

### 2.7. Ethical Considerations

Ethical approval was received from the University of the Free State (UFS) Health Science Research Ethics Committee (UFS-HSREC) with the ethical approval number UFS-HSD2019/1129/2910. Further approval to perform the study was received from the Free State Department of Health, Bloemfontein, South Africa. An information document with a clear description of the study in the language of choice was provided for the parent/guardian. Permission and consent in the language of choice was also requested from the parent/guardian for their children to participate in the study.

## 3. Results

### 3.1. General Information, Demographics, and Clinical Presentations

Of the participants enrolled, a total of n = 28 were recruited during each of the three periods investigated. Demographically, 34 (40.4%) were females, while 50 (59.6%) were males. The age range of participants was from 0 to 60 months, with most patients (57, 67.8%) aged 0–24 months. Of the participants, 35 (41.6%) required oxygenation, and 17 (20%) had human immunodeficiency virus (HIV), 24 (28.5%) had chest indrawing, 9 (10.7%) had feeding difficulty, 3 (3.5%) were asthmatic, and 0 (0%) required the need for intensive care unit.

### 3.2. Detected Pathogens and Co-Infections

**Figure 1:**
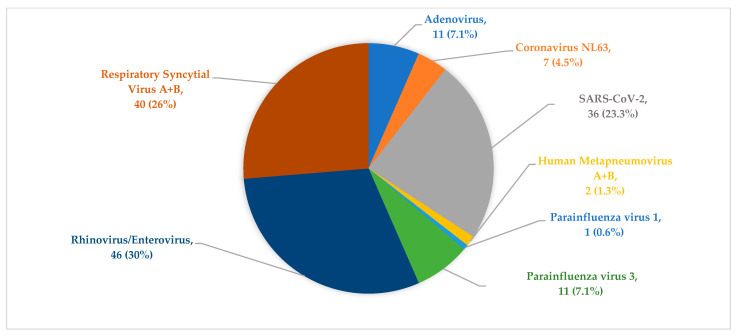
Profile of pathogens detected in all 74 positive samples across the three periods reviewed (during and between the second and third wave of the COVID-19 pandemic) depicting the percentage of detection frequency of each virus. SARS-CoV-2 = severe acute respiratory syndrome coronavirus 2.

Of the 21 pathogens targeted by the QIAstat-Dx Respiratory SARS-CoV-2 Panel, across all participants, 8/21 viral pathogens were detected. Of the 84 participants recruited, n = 74 (88%) tested positive for at least one viral pathogen, and n = 10 (12%) cases had no targeted pathogens detected. Of the detected viral pathogens, HRV/enterovirus (EVs) (n = 46 (30%)), RSV A+B (n = 40 (26%)), and SARS-CoV-2 (n = 36 (23.3%)) were the most detected pathogens (Figure 1). Other viral pathogens detected were PIV 3 (n = 11 (7.1%)), adenovirus (Adv) (n = 11 (7.1%)), coronavirus NL63 (n = 7 (4.5%)), HMPV (n = 2 (1.3%)), and PIV 1 (n = 1 (0.6%)). The percentage of detection frequency is depicted in Figure 1. None of the three targeted bacteria pathogens were detected. Amongst the participants that tested positive, single, double, triple, and quadruple viral infections were detected in n = 20, n = 31, n = 19, and n = 4 children, respectively (Table 2). Full data are provided in Supplementary Data Table S1.

In the 20 non-co-infected samples, detected pathogens were RSV (n = 8), SARS-CoV-2 (n = 5), HRV/EVs (n = 5), Adv (n = 1), and HMPV (n = 1) (Table 2). Of the 54 co-infected samples, the most frequent combinations of pathogens were RSV + HRV/EVs (n = 9/54), HRV/EVs + SARS-CoV-2 (n = 7/54), and HRV/EVs + SARS-CoV-2 + RSV (n = 7/54) (Table 2). The most detected viral agents in single and co-infection cases were RSV, SARS-CoV-2, and HRV/EVs. However, PIV 3, PIV 1, and coronavirus NL63 were only found in co-infections. The abundance of nucleic acid in the respiratory specimens obtained from these patients was diverse, with Ct values between 15.3 and 37.9 (Table 2). Full data are provided in Supplementary Data Table S1.

### 3.3. Evaluation of Clinical Symptoms with Viral Co-Infection

**Figure 2:**
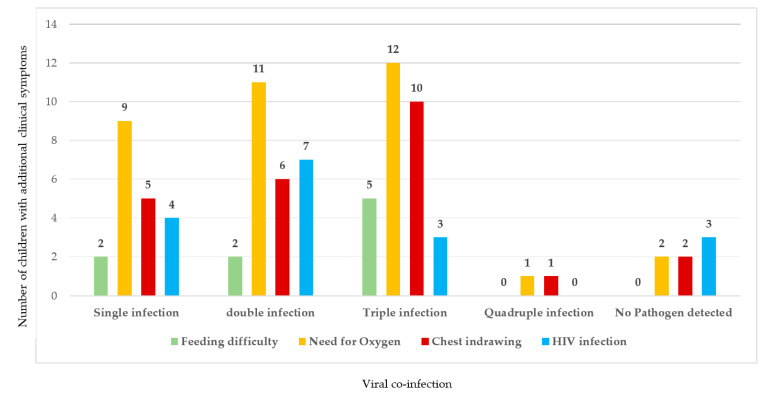
Estimate of clinical symptoms versus viral co-infections.

The severity of additional clinical symptoms is not directly associated with higher number of co-infecting viruses, as shown in Figure 2. Moreover, HIV infection was not significantly associated with increased viral co-infection rate, considering that 4/17 (23%), 7/17 (41%), and 3/17 (18%) children with HIV infection presented with single, double, and triple viral co-infection, respectively. Moreover, none presented with quadruple infection and 3/17 (18%) had no pathogen detected. In addition, 6/17 (35%) children with HIV had chest indrawing and need for oxygen, 7/17 (41%) presented with neither of the two symptoms, 4/17 (24%) had need for oxygen but with no chest indrawing, and none had feeding difficulty (Figure 2 and Table S1).

### 3.4. Profile of Pathogens Detected during Each Wave of the COVID-19 Infection

**Figure 3:**
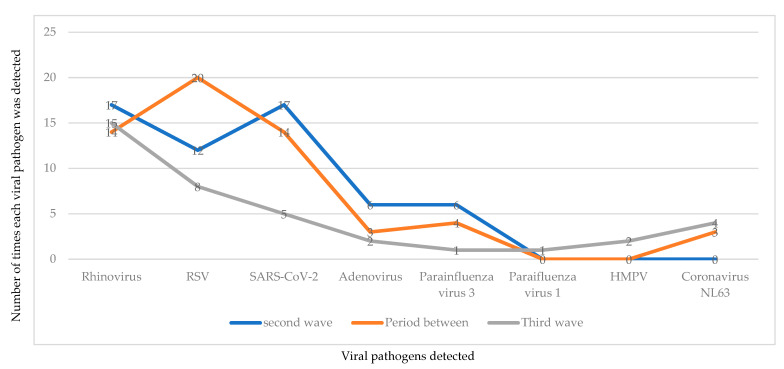
A graphical description on the number of times each viral pathogen was detected across the three study periods (during and between the second and third wave of COVID-19 infection). RSV=respiratory syncytial virus, SARS-CoV-2 = severe acute respiratory syndrome coronavirus 2, HMPV = human metapneumovirus.

Of the 28 children included during each period (second wave, the period between, and the third wave of the COVID-19 infection), a total of n = 26, n = 26, and n = 22 children tested positive for at least one viral pathogen, respectively, and no targeted pathogens were detected in n = 2, n = 2, and n = 6 children, respectively. The profile of pathogens detected during each period investigated are depicted in Figure 3.

## 4. Discussion

Globally, viral aetiology of SARI varies between 2 and 80% [14,37,38,39,40,41,42]. In this study, the QIAstat-Dx Respiratory SARS-CoV-2 Panel (Qiagen Hilden, Germany), which has been critically evaluated [32,33,36,43], was used for respiratory pathogen detection. A viral aetiology was found in 74/84 (88%) of hospitalised patients. In similar studies, the viral detection rate ranged from 50 to 80%. For instance, a study by Wishaupt et al. had 457/560 (81.6%) positivity rate using an in-house RT-PCR to test for 15 viruses and 2 bacteria from children ≤12 years [40]. A recent study used xMAP multiplex assays to test for 13 known respiratory viruses from NPS taken from 4880 children ≤13 years in China. At least one viral pathogen was detected in 77.1% of the samples tested [41]. Another study used the FTD Respiratory Pathogens 21 plus to screen the NPS samples collected from children and detected >80% of viruses in the samples [42].

Similar to other studies having cases of SARI/ARI with no targeted pathogens detected [10,40,41,44], a total of n = 10 (12%) of the SARI cases in this study had no targeted pathogen detected. This study also showed zero roles for atypical bacteria (0%) in causing SARI. Suggestively, this may be due to the causative viral/bacteria pathogen not targeted by the QIAstat-Dx Respiratory SARS-CoV-2 Panel, or the pathogen could be present but below the assay’s limit of detection. It could also be due to a lower respiratory infection that could have been missed through upper respiratory sampling. However, this can be evaluated using metagenomics next-generation sequencing (mNGS), which may yield further results.

The unprecedented COVID-19 disease, caused by SARS-CoV-2, has become a global pandemic with severe health consequences [45]. Several countries have experienced two to four infection waves [46,47,48,49]. In South Africa, a three-wave pattern had been observed during the period of this study. Although children (aged less than five years) do not bear the health burden of COVID-19, they may not be spared from its consequences [50]. The NPIs adopted to contain the spread of COVID-19 infection had thankfully decreased the spread of SARS-CoV-2. However, it has also concurrently altered the spectrum of other common viruses, such as RSV and IFV [28,51]. This, in effect, could leave children more vulnerable/susceptible to other respiratory viruses with increased morbidity [31,51].

Notably, fewer viral pathogens were detected during the third wave of the COVID-19 infection, and this could be due to stricter adherence to the implemented COVID-19 prevention strategies (NPIs) during the period between second and third waves, which may have resulted in low pockets of viral circulation during the third wave of infection. The unchanged detection rate for HRV is not surprising, as HRV is often detected throughout the year in South Africa [28,52,53,54]. Moreover, its relative resistance to ethanol-containing disinfectants and survivability on surfaces for prolonged periods [55,56] could have contributed to its resistance to NPIs [31,52]. Reduced IFV detection and increased RSV detection in the year 2020 post-COVID-19 inception have also been reported in South Africa [43] and in facility-based surveillance from Australia [57]. Significantly lower/almost non-existence of IFV detection was reported in Japan, Korea, and the USA during the COVID-19 pandemic [53,58,59]. Comparatively, there are some contrasting findings between the present study and other similar studies where a near/complete disappearance of IFV and RSV was reported during the COVID-19 pandemic [28,31,52,53,60]. It was suggested that travel restrictions may have affected the circulation of IFV rather than RSV due to their more defined seasonality in South Africa [51]. Moreover, RSV is detected throughout the year, with a surge in the first half, thus maintaining local reservoirs [51]. In addition, the transmissibility characteristics of IFV vs. RSV could have been impacted by the COVID-19 control measures. This is because influenza has a lower effective reproductive number (R) (R: 1.2–1.4) [61] compared to RSV (R: 1.7–2.1) [62], which could have resulted in a more profound effect on IFV compared to RSV [51]. Moreover, viral interference between influenza A virus (IFV-A) and HRV has been documented [63,64].

Whilst children have been proposed to be less susceptible to COVID-19 infection, it does not negate that they can still become infected. This notion was substantiated in the present study, with the SARS-CoV-2 virus detected in 36/84 patients (31 presented as co-infections and 5 presented as single infections), all of whom required hospitalisation. Moreover, some studies have reported possible transmission from children [65,66]. In effect, this may necessitate the continued surveillance and isolation, if possible, of SARS-CoV-2 cases in children towards adequate infection control.

As previously shown, mRT-PCR methods enable sensitive detection of viral co-infections [8,67,68]. In this study, viral co-infection had a proportion of 64%, with RSV/HRV-EVs/SARS-CoV-2 being the most frequent combination. Previous findings showed that the proportion of viral co-infections in children ranged from 4.0 to 69%, where RSV, HRV, and IFV were mostly implicated [8,13,67,68,69]. Overlapping of viral epidemic seasons and increased single infection rate of particular viruses are factors that may influence the prevalence of co-infection [8]. Currently, data supporting the clinical severity of co-infection versus single infection are controversial [70,71,72,73]. The health outcome of viral co-infection may also vary depending on the pathogen implicated, as well as the viral load [72,73]. Although some studies documented factors such as prolonged mechanical ventilation, need for intensive care unit (ICU), and lengthy hospitalisation in co-infected children [74], other studies reported little or no difference between children with single vs. co-infection in terms of the stated factors [44,75,76]. In this study, viral co-infection was not strongly associated with worse clinical symptom presentations. Although there are more children who presented with triple viral respiratory co-infection with feeding difficulty, chest indrawing, and need for oxygen, nonetheless, there are children with quadruple viral respiratory co-infection without any of the stated symptoms.

Furthermore, children with HIV have been reported to have a higher frequency and worse prognosis with viral respiratory infections [77,78]. Notably, severe forms of RTIs are particularly heightened in HIV positive children who are not on antiretroviral therapy [79,80]. In this regard, some studies have reported increased co-infection and high case fatality rate in children with HIV compared to HIV-uninfected children [81,82,83]. On the contrary, a study by Majozi and colleagues documented similar mortality rates between these groups [84]. In this study, the proportion of HIV-infected children in the participants was 20.2% (17/84), and HIV was not significantly associated with extreme clinical symptoms or increased viral co-infection rate. As noted, none of the HIV-infected children had feeding difficulty and only 6/17 had chest indrawing and need for oxygen. Moreover, 3/17 of the HIV-infected children presented with triple co-infection, and none presented with quadruple co-infection. Comparatively, a significant number (20/67) of the HIV-uninfected children had triple–quadruple viral co-infections. It can be suggested that the lesser detection of viral co-infection and lower percentage of reported severe symptoms presentations in the HIV-infected children in this study could be due to the relatively low numbers of this group in the participants. Nonetheless, further research into the clinical dynamics of viral co-infections in HIV-infected and uninfected children is still needed for an improved knowledge of host–virus interactions.

Some limitations of this study should be acknowledged. Firstly, due to this study’s restriction to nasopharyngeal/oropharyngeal samples, it is possible that it overlooked a relationship between lower airway carriage of bacteria and SARI hospitalisation. Another limitation is that the length of hospital stay and details of death, if any, were not recorded for this study. Therefore, the consequences of co-infection, the effect of asthma, the requirement for oxygenation, or HIV infection on the clinical course/outcome could not be established. This study was also limited by sample size due to the limited number of available QIAstat-Dx cartridge for diagnosis. However, it has provided baseline data on the spectrum of viruses circulating in children ≤5 years during the different waves of COVID-19 infection in the country while simultaneously highlighting viruses as a significant contributor to SARI morbidity in the study setting.

## 5. Conclusions

It is becoming more crucial to make a prompt and accurate identification of respiratory infections in clinical settings in order to prevent the spread of virulent or resistant microorganisms [85,86], especially in an era of pandemic, such as COVID-19. This study demonstrated a high detectability of mRT-PCR for the targeted viral respiratory pathogens, indicating that mRT-PCR-based techniques may be useful as a possible point-of-care test in timely detection of viral agents. Additionally, this study provided a first overview of viral agents present in children hospitalised with SARI during and between the second and third waves of COVID-19 infection in South Africa and identified changes in viral spectrum during the COVID-19 pandemic. The total absence of virus, such as influenza and disruption of known/normal circulation patterns of other respiratory viruses during this period is attributed to NPIs against transmission of COVID-19. These changes in viral circulation spectrum could result in waned immunity and increased susceptibility to SARI post NPIs relaxation. Therefore, as most NPIs are being relaxed, the implications of this relaxation for future outbreaks of other viral RTI should be considered, since susceptibility would have built up over the NPIs period. Consequently, there may be need for future epidemiological surveillance to evaluate and monitor the dynamics of other viral respiratory pathogens post-COVID-19 NPIs relaxation. Although, the strikingly high viral co-infection rate is of concern, as it may complicate clinical decision, especially in an era of COVID-19. Nonetheless, the accurate detection and reporting of causative/co-infecting viral pathogens in SARI cases may be imperative towards outbreak identification and epidemiological controls. Additionally, the absence of pathogens in 12% cases and absence of bacteria aetiology in this cohort can be further evaluated using metagenomic next-generation sequencing. To the best of our knowledge, this is the first study in South Africa to report on the viral profile of respiratory pathogens in children ≤5 years admitted with SARI during and between the second and third waves of COVID-19 infection in the country.

## Figures and Tables

**Table 1:** Period of different COVID-19 infection waves as experienced in South Africa.

Waves	Second Wave	Period between	Third Wave
Timeline	December 2020–February 2021	February–April 2021	June 2021–August 2021
Definition	Period with greater than 30 cases per 100,000 persons	Period between second and third waves with fewer than 30 cases per 100,000 persons	Period with greater than 30 cases per 100,000 persons

**Table 2:** Pathogens detected in single and co-infections and detected Ct values.

Viral Pathogens Detected	No. of Times Pathogens Was/Were Detected	Average Ct Values ^a^	Standard Deviation ^b^
**Single viral infections** RSV HRV HMPV SARS-CoV-2 Adv	8 5 1 5 1	24.1 27.9 34.3 35.3 31.1	4.84 1.95 n/a 0.9 n/a
**Double viral co-infections** RSV + SARS-CoV-2 HRV/EVs + SARS-CoV-2 HRV/EVs + RSV RSV + Adv SARS-CoV-2 + Adv SARS-CoV-2 + PIV 3 PIV 3 + HRV/EVs RSV + Coronavirus NL63 Coronavirus NL63 + HRV/EVs HMPV + HRV/EVs HRV/EVs + Adv PIV 1 + RSV	4 7 9 1 1 1 1 3 1 1 1 1	25.6 & 33.4 29.0 & 33.6 30.9 & 28.5 30.6 & 34.1 32.5 & 33.4 37.9 & 33.3 26.3 & 33.4 28.7 & 32.3 34.8 & 32.5 31.1 & 30.3 27.6 & 32.3 33.1 & 25.6	5.0 & 3.3 2.62 & 1.52 2.4 & 4.40 n/a n/a n/a n/a 1.67 & 1.37 n/a n/a n/a n/a
**Triple viral co-infections** SARS-CoV-2 + PIV 3 + RSV PIV 3 + HRV/EVs + SARS-CoV-2 HRV + Adv + SARS-CoV-2 PIV 3 + HRV/EVs + Adv SARS-CoV-2 + RSV + HRV/EVs RSV + HRV/EVs + PIV 3 SARS-CoV-2 + RSV + Adv Coronavirus NL63 + PIV 3 + HRV/EVs Coronavirus NL63 + RSV + HRV/EVs Coronavirus NL63 + HRV/EVs + Adv	1 3 2 1 7 1 1 1 1 1	32.5 & 28.9 & 21.1 31.0 & 30.7 & 33.8 33.3 & 31 & 34.1 31.3 & 27.6 & 33.7 34.7 & 26.5 & 32 26.5 & 32.0 & 31.0 35.1 & 30.6 & 35.7 36.2 & 32.0 & 33.7 34.2 & 28.6 & 34.4 29.1 & 30.6 & 35.7	n/a 1.38 & 1.8 & 0.75 1.9 & 4.7 & 1.1 n/a 0.9 & 3.1 & 1.76 n/a n/a n/a n/a n/a
**Quadruple viral co-infections** SARS-CoV-2 + RSV + HRV/EVs + PIV 3 SARS-CoV-2 + RSV + Adv + HRV/EVs SARS-CoV-2 + Adv + HRV/EVs + PIV3	2 1 1	34.9 & 30.3 & 30.4 & 29.3 35.3 & 26.5 & 35.1 & 31.4 34.5 & 35.7 & 32.2 & 27.8	1.3 & 3.45 & 1.1 & 3.2 n/a n/a

RSV = respiratory syncytial virus, HRV = rhinovirus, SARS-CoV-2 = severe acute respiratory syndrome coronavirus 2, Adv = adenovirus, PIV 3 = parainfluenza virus 3, PIV 1 = parainfluenza virus 1, HMPV = human metapneumovirus, EVs = enterovirus. ^a^ Average Ct values are presented for pathogens detected more than once. Actual Ct values are written for those detected once. ^b^ Standard deviations are presented for pathogens detected more than once.

## Data Availability

The data presented in this study are available in Supplementary Table S1.

## Supplementary Materials

The following supporting information can be downloaded at: https://www.mdpi.com/article/10.3390/ijerph191610418/s1. Data supplementary to individual participants in the study are provided in Table S1.
