# Supplementary material for: Pathogen Profile of Children Hospitalised with Severe Acute Respiratory Infections during COVID-19 Pandemic in the Free State Province, South Africa
Source: Int J Environ Res Public Health. 2022 Aug 21;19(16):10418. doi: 10.3390/ijerph191610418 (PMC9408356; doi:10.3390/ijerph191610418)
Supplement: Supplementary file 1 [file ijerph-19-10418-s001.zip › ijerph-1777982-supplementary.pdf]

Table S1. Pathogen and clinical profile of individual participants included in the study.

Sample No	Record No	Age	Gender	Feeding difficulty	HIV Infected	Need for oxygen	Chest Indrawing	ICU Admission	Asthmatic	Viruses detected using Qiastat and Ct Values
1.	A301	2 years 5 months	Female	No	No	Yes	Yes	No	No	Rhinovirus/Enterovirus (31.4), Adenovirus (35.7), SARS-CoV-2 (35.2)
2.	Z55	1 Year 4 Months	Male	No	No	No	No	No	No	Rhinovirus/Enterovirus (33.5), RSV A+B (25.3), SARS-CoV-2 (35.5)
3.	Z11	7 months	Male	No	No	No	No	No	No	Rhinovirus/Enterovirus (31.9), RSV A+B (27.7)
4.	A29	11 months	Male	No	No	Yes	Yes	No	No	Rhinovirus/Enterovirus (28.1)
5.	A34	2 weeks	Female	Yes	No	Yes	Yes	No	No	Rhinovirus/Enterovirus (34.3), RSV A+B (24.1), SARS-CoV-2 (35.5)
6.	Z26	4 months	Male	No	Yes	Yes	Yes	No	No	Rhinovirus/Enterovirus (30.8)
7.	Z58	1 Month	Female	No	No	No	No	No	No	Rhinovirus/Enterovirus (26.5), RSVA+B (25.5)
8.	Z51	1 Month 3 weeks	Female	No	No	Yes	Yes	No	No	Parainfluenza virus 3 (31.0), Rhinovirus (32.0), Rsv A+B (26.5)
9.	Z38	1 year 2 weeks	Female	No	Yes	No	No	No	No	SARS-CoV-2 (36.4)
10.	Z7	3 years 8 months	Male	No	No	No	No	No	No	SARS-CoV-2 (34.8)
11.	Z34	1 year 9 months	Male	No	Yes	Yes	Yes	No	No	RSV A+B (26.0)SARS-CoV-2 (34.4).
12.	Z32	1 year 4 months	Male	No	No	Yes	Yes	No	No	RSV A+B (29.4)
13.	Z49	4 Months	Female	No	No	No	No	No	No	RSV A+B (15.3)
14.	Z75	2 years 3 Months	Male	Yes	No	Yes	No	No	No	SARS-CoV-2 (35.2)
15.	Z27	3 months	Male	No	No	No	No	No	No	RSV A+B (19.4), SARS-CoV-2 (32.9)
16.	Z44	4 years 6 months	Male	No	Yes	Yes	Yes	No	No	Negative for All Viral agents
17.	Z37	1 year 6 months	Male	No	No	No	No	No	No	Rhinovirus (29.1), RSV A+B (20.8)
18.	Z67	9 Months	Female	No	No	No	No	No	No	Parainfluenza virus 3 (26.3), Rhinovirus (33.4).

19.	W43	1 Year 8 months	Male	No	No	No	No	No	No	Negative for All Viral agents
20.	Z9	4 years 4 months	Female	No	Yes	No	No	No	No	Rhinovirus (31.4), SARS Cov 2 (33.3), RSV A+B (30.2)
21.	Z23	1 month 2 weeks	Male	No	Yes	Yes	No	No	No	Coronavirus NL63 (34.2), RSV A+B (30.8)
22.	Z15	1 years 10 months	Male	No	No	No	No	No	No	Rhinovirus (32.9), RSV A+B (27.4), SARS CoV 2 (34.6)
23.	W6	2 Months	Male	No	Yes	Yes	No	No	Yes	SARS CoV 2 (34.0)
24.	W26	4 Years 3 months	Female	No	Yes	Yes	No	No	No	Coronavirus NL63 (34.8), Rhinovirus/Enterovirus (32.5)
25.	W33	4 Years 4 months	Female	No	Yes	No	No	No	No	Negative for All Viral agents
26.	W12	1 Years 9 months	Male	No	No	No	No	No	No	Rhinovirus/Enterovirus (28.3), RSV A+B (22.9)
27.	W3	10 Months	Male	No	No	Yes	Yes	No	No	Rhinovirus/Enterovirus (32.5), RSV A+B (30.1)
28.	W24	9 Months	Male	No	No	Yes	Yes	No	No	Rhinovirus/Enterovirus (30.0)., RSV A+B (30.7)
29.	W43	1 year 6 months	Male	No	No	No	No	No	No	SARS-CoV-2 (36.2)
30.	Z35	3 months	Male	No	Yes	Yes	Yes	No	No	SARS-CoV-2 (35.1), RSV A+B (30.6), Adenovirus (35.7)
31.	Z45	1 year 1 month	Female	Yes	No	Yes	Yes	No	No	RSV A+B (28.7), Coronavirus NL63 (32.0)
32.	W16	4 years 8 months	Male	No	No	No	No	No	No	Rhinovirus (27.6)
33.	W34	11 Months	Male	No	No	No	No	No	No	Rhinovirus (30.3), Human metapneumovirus (31.1)
34.	W41	2 Years 3 months	Male	No	No	No	No	No	No	Coronavirus NL63 (29.1), Rhinovirus/Enterovirus (30.6), Adenovirus (35.7)
35.	W40	1 month 1 week	Female	No	No	Yes	No	No	No	Human metapneumovirus (34.3)
36.	W44	2 Years 4 months	Female	Yes	No	Yes	No	No	No	Rhinovirus (24.7)
37.	W42	11 months	Male	No	Yes	No	No	No	No	Rhinovirus (27.6), Adenovirus (32.3)

38.	W27	4 Years 2 months	Male	No	No	No	No	No	No	SARS-CoV-2 (33.6), Rhinovirus/Enterovirus (29.1)
39.	W19	11 months	Male	No	No	No	No	No	No	RSV A+B (21.3)
40.	W38	8 Months	Male	No	Yes	Yes	No	No	No	RSV A+B (25.6) Parainfluenza virus 1 (33.1)
41.	W32	3 Years 2 months	Female	No	No	No	No	No	No	Negative for All Viral agents
42.	W22	3 Years 4 Months	Female	No	No	No	No	No	No	Negative for All Viral agents
43.	W31	4 Years 4 months	Female	No	No	No	No	No	No	Rhinovirus (28.5)
44.	W10	6 Months	Male	No	Yes	Yes	Yes	No	Yes	SARS-CoV-2 (33.6), Rhinovirus/Enterovirus (28.5), RSV A+B (29.5)
45.	A39	1 year 2 months	Female	Yes	No	Yes	No	No	No	SARS-CoV-2 (32.5), Parainfluenza virus 3 (28.9), RSV A+B (21.1)
46.	A28	1 year 11 months	Male	No	No	No	No	No	No	RSV A+B (29.9)
47.	Z24	10 months	Female	No	No	No	No	No	No	RSV A+B (30.6), Adenovirus (34.1)
48.	A25	4 years 5 months	Female	No	No	No	No	No	No	Negative for All Viral agents
49.	A33	2 months	Male	No	No	Yes	Yes	No	No	Parainfluenza virus 3 (31.3), Rhinovirus (27.6), Adenovirus (33.7)
50.	A23	5 months	Male	No	No	Yes	Yes	No	No	SARS-CoV-2 (35.4), RSV A+B (20.7), Rhinovirus (32.3)
51.	A32	1 year 8 months	Male	No	No	No	No	No	No	SARS-CoV-2 (36.2), RSV A+B (26.9), Rhinovirus/Enterovirus (29.3),Parainfluenza virus 3 (33.0)
52.	A27	3 years 4 months	Female	Yes	No	Yes	No	No	No	SARS-CoV-2 (33.7), Rhinovirus/Enterovirus (24.3)
53.	A30	2 years 2 months	Female	No	No	Yes	Yes	No	No	SARS-CoV-2 (33.6), Parainfluenza virus 3 (32.3), Rhinovirus (29.3)
54.	A26	1 months	Male	Yes	No	Yes	Yes	No	No	SARS-CoV-2 (33.1), Parainfluenza virus 3 (29.1), Rhinovirus (29.7)
55.	A35	2 years 9 months	Male	No	No	No	No	No	No	RSV (33.4), SARS-CoV-2 (28.7)
56.	A31	3 months	Male	No	Yes	No	No	No	No	Rhinovirs/Enterovirus (31.0), SARS-CoV-2 (32.8)

57.	A38	3 years 2 months	Female	No	No	No	No	No	No	Adenovirus (33.4), SARS-CoV-2 (32.5)
58.	Z606	2 years 10 months	Female	No	No	No	No	No	No	RSV A+B (22.6)
59.	A40	1 year 3 months	Male	Yes	No	No	No	No	No	Rhinovirus/Enterovirus (26.7), SARS-CoV-2 (34.1)
60.	W1	3 months	Male	No	No	Yes	Yes	No	No	Rhinovirus (31.4), Adenovirus (35.1), RSV A+B (26.5), SARS-CoV-2 (35.3)
61.	Z28	4 years 8 months	Female	No	No	Yes	No	No	Yes	Rhinovirus/Enterovirus (31.1), RSV A+B (28.7), SARS-Co-V (35.5)
62.	Z53	11 months	Male	No	No	No	No	No	No	Parainfluenza virus 3(25.6), Rhinovirus/Enterovirus (31.5), RSV A+B (33.8), SARS-CoV-2 (33.6)
63.	Z76	3 Months	Male	No	No	Yes	Yes	No	No	RSV A+B (28.5)
64.	Z78	8 months	Female	No	No	No	No	No	No	Parainfluenza virus 3 (33.3), SARS-CoV-2 (37.9)
65.	Z16	16 months	Female	No	No	No	No	No	No	RSV A+B (31.5), Rhinovirus/Enterovirus (33.8)
66.	W14	6 Months	Male	No	Yes	Yes	Yes	No	No	Negative for All Viral agents
67.	W8	13 Months	Male	No	No	Yes	No	No	No	RSV A+B (32.8), Rhinovirus/Enterovirus (32.4)
68.	W7	16 Months	Female	No	No	No	No	No	No	Coronavirus NL63 (36.2), Parainfluenza virus 3 (32.0), Rhinovirus/Enterovirus (33.7)
69.	W29	1 Year 8 Months	Male	No	No	Yes	Yes	No	No	Adenovirus (31.1)
70.	W13	10 Months	Male	No	No	Yes	Yes	No	No	Coronavirus NL63 (30.9), RSV A+B (26.7)
71.	W28	11 Months	Female	No	No	No	No	No	No	Coronavirus NL63 (34.2), RSV A+B (28.6), Rhinovirus/Enterovirus (34.4)
72.	W11	2 years 2 months	Female	No	No	No	No	No	No	Rhinovirus/Enterovirus (28.6), SARS-CoV-2 (30.8)
73.	W36	1 Year 1 month	Male	No	No	No	No	No	No	Negative for All Viral agents
74.	Z60	1 Year 2 months	Male	No	No	No	No	No	No	Parainfluenza virus 3(27.8), Rhinovirus/Enterovirus (32.3), Adenovirus (35.7), SARS-CoV-2 (35.3)

75.	Z1	3 Years 5 months	Male	No	No	No	No	No	No	Negative for All Viral agents
76.	Z21	1 year 1 month	Female	Yes	No	Yes	Yes	No	No	Parainfluenza virus 3(31.7), Rhinovirus/Enterovirus (33.3), SARS-CoV-2 (34.9)
77.	A1	1 year 9 months	Male	No	No	No	No	No	No	Rhinovirus/Enterovirus (35.2) Adenovirus (26.3), SARS-CoV-2 (33.0)
78.	Z20	5 months	Female	No	Yes	No	No	Unspecified	No	RSV A+B (23.8) SARS-CoV-2 (37.9)
79.	Z19	1 year 4 months	Male	No	No	No	No	No	No	RSV A+B (20.3)
80.	Z3	3 years 7 months	Male	No	No	No	No	No	No	Rhinovirus/Enterovirus (32.6), SARS-CoV-2 (36.3)
81.	Z5	5 months	Female	No	No	No	No	No	No	Rhinovirus/Enterovirus (33.6) RSV A+B (34.8)
82.	Z6	2 years 10 months	Female	No	Yes	No	No	No	No	RSV A+B (26.2)
83.	A37	4 years 1 month	Male	No	No	No	No	No	No	Negative for All Viral agents
84.	A36	3 years 2 months	Female	No	No	Yes	Yes	No	No	Rhinovirus/Enterovirus (30.9), SARS-CoV-2 (34.2)
